# New Insights on Mechanisms and Therapeutic Targets of Cerebral Edema

**DOI:** 10.2174/1570159X22666240528160237

**Published:** 2024-05-29

**Authors:** Pei Shang, Ruoyi Zheng, Kou Wu, Chao Yuan, Suyue Pan

**Affiliations:** 1 Department of Neurology, Nanfang Hospital, Southern Medical University, Guangzhou, China;; 2 Department of Neurology, Mayo Clinic College of Medicine and Science, Rochester, MN, USA

**Keywords:** Cerebral edema, glymphatic system, intracranial pressure, blood-brain barrier, meningeal lymphatic vessels, mannitol

## Abstract

Cerebral Edema (CE) is the final common pathway of brain death. In severe neurological disease, neuronal cell damage first contributes to tissue edema, and then Increased Intracranial Pressure (ICP) occurs, which results in diminishing cerebral perfusion pressure. In turn, anoxic brain injury brought on by decreased cerebral perfusion pressure eventually results in neuronal cell impairment, creating a vicious cycle. Traditionally, CE is understood to be tightly linked to elevated ICP, which ultimately generates cerebral hernia and is therefore regarded as a risk factor for mortality. Intracranial hypertension and brain edema are two serious neurological disorders that are commonly treated with mannitol. However, mannitol usage should be monitored since inappropriate utilization of the substance could conversely have negative effects on CE patients. CE is thought to be related to blood-brain barrier dysfunction. Nonetheless, a fluid clearance mechanism called the glial-lymphatic or glymphatic system was updated. This pathway facilitates the transport of cerebrospinal fluid (CSF) into the brain along arterial perivascular spaces and later into the brain interstitium. After removing solutes from the neuropil into meningeal and cervical lymphatic drainage arteries, the route then directs flows into the venous perivascular and perineuronal regions. Remarkably, the dual function of the glymphatic system was observed to protect the brain from further exacerbated damage. From our point of view, future studies ought to concentrate on the management of CE based on numerous targets of the updated glymphatic system. Further clinical trials are encouraged to apply these agents to the clinic as soon as possible.

## 1 INTRODUCTION

Stated by Monro-Kellie doctrine is that the intracranial constituent enclosed by the non-expandable skull has a fixed volume and contains three compartments, including intracranial blood in the arterial and venous compartments, brain tissue, and Cerebrospinal Fluid (CSF), whose volume remains nearly constant after the closure of the fontanelles [1]. In pathological conditions, increases in any volume would result in substantial elevations of Intracranial Pressure (ICP), which might cause serious repercussions. Many neurological conditions, such as traumatic brain injury (TBI), stroke, intracranial hemorrhage, intracranial infection, hydrocephalus, and brain tumors, can result in ICP elevation as a consequence [2]. Clinically, patients with elevated ICP frequently exhibit headaches, nausea, vomiting, loss of consciousness, and papilledema. If intervention is delayed, a secondary head injury may develop. Mechanically, three key intracranial processes, including abnormal CSF dynamics such as CSF hypersecretion, reduced CSF outflow over the arachnoid granulations or lymphatics, and elevated venous sinus pressure, have been identified as contributing factors to elevated ICP [3]. Traditionally, Cerebral Edema (CE), which is associated with obstruction of CSF drainage, has also been suggested to result in raised ICP.

A wide range of Central Nervous System (CNS) problems, such as brain trauma, tumors, strokes, infections, metabolic disorders, and others, are greatly exacerbated by CE, a pathological increase in the water mass retained by the brain interstitial space [4]. Evidence for diagnosis and differentiating various types of CE is provided by the combination of clinical symptoms and imaging techniques like CT or MRI: While Vasogenic Edema (VasE) disproportionately affects subcortical white matter with relative sparing of the cerebral cortex and subcortical gray matter, the early Cytotoxic Edema (CytE) adversely affects both gray matter and white matter components, resulting in loss of cortical-subcortical distinction [5]. With mild edema, increased brain volume is compensated for by decreasing CSF and blood volume, while rapidly progressive edema overwhelming cerebral autoregulatory mechanisms will result in structural compression, cerebral ischemia, and even ultimately, fatal cerebral herniation generated. The outcome of malignant CE is nearly catastrophic, with a death rate of close to 80%: massive cerebral edema, increased ICP that followed, quick decline in neurological function, and transtentorial herniation [6]. Clinically, approaches such as ventilation, hypothermia, hyperosmolar therapy, and decompressive craniectomy have been used to evaluate and treat CE using intracranial hypertension as a stand-in for CE. However, it is concerned that these treatments might not be suitable for all patients with CE since ICP is less likely to be a complete reflection of CE, and side effects of these treatments may cause poor prognosis. This underscores the necessity of developing targeted therapies to expand the treatment armamentarium for CE by focusing on the underlying molecular and cellular mechanisms of edema formation.

Under peripheral circumstances, fluid normally circulates freely between the interstitial space and plasma, returning mostly *via* the lymphatic system. Diseases and drugs have the potential to upset this equilibrium, resulting in an excessive flow of fluid out of the vascular space or an impaired lymphatic return of fluid from the interstitial space, which causes edema. However, CSF in high amounts can enter the brain quickly and cause tissue swelling by glymphatic exchange with the Interstitial Fluid (ISF) [7], which indicates that edema in the CNS system might be different from that in the periphery. Mestre *et al.* discovered that the primary mechanism for early edema production and ion perturbation during ischemic stroke is CSF entrance into the brain by the glymphatic channel [8], which sheds novel light on the mechanism of CE formation. To understand how edema develops in the brain, two features must be identified: the driving force, which “pushes” substances into the brain, and the permeability pore, which permits a transcapillary passage of these substances from the intravascular to the extracellular space. This basic principle was established by Starling more than a century ago [9]. Edema will occur if this physiological balance that controls the fluid is upset under abnormal circumstances. Additionally, Tight Junctions (TJs) between Endothelial Cells (ECs) may become dysfunctional under pathological conditions, destroying their equilibrium, and the altered fluid transport will contribute to edema [9].

The progression of CE occurs in three distinct phases separately over time and space: the CytE phase, the subsequent ionic edema phase, and the most severe VasE phase. CytE, characterized by the swelling of astrocytes and neuronal dendrites, occurs within minutes after ischemic insult without Blood-Brain Barrier (BBB) destruction, which is usually the consequence of ATP consumption and the subsequent disruption of cell membranes, providing opportunities for water to shift from the extracellular to the intracellular compartment [6]. At the same time, it gives rise to the ionic gradient between the vascular compartment and ISF, which acts as a catalyst for later ionic and vasogenic edema and causes swelling of the brain tissue [6]. VasE is defined by the ability of water, electrolytes, blood, and plasma proteins, such as albumin and IgG, to leak into the brain interstitial compartment once the BBB becomes dysfunctional [10, 11]. As a result, CE processes and CE category definitions are closely related to the integrity of BBB.

Since the brain's lymphatic drainage system was only recently discovered, research on the causes of CE has primarily focused on BBBs. However, the 2015 discovery of the classical lymphatic drainage system in the dura mater of the brain requires a reevaluation of CE development and provides new insight into the causes of the neuroinflammatory mechanisms that lead to BBB breakdown [12]. The capability of the lymphatic drainage system not only lies in absorbing CSF from the adjacent subarachnoid space but also in providing the pathway for both the entrance and exit of immune cells from the CNS. To thoroughly explore the relationship between CE and intracranial hypertension and, as a result, to generate better patent treatments for CE resolution in the future, we reviewed the role of the updated lymphatic system played in both CE creation and protection from further aggregation in our study.

## 2 BASIC FACTORS FOR CEREBRAL EDEMA FORMATION

### 2.1 Blood-brain Barrier

The BBB is a term that is described to have unique properties that allow the continuous, nonfenestrated CNS vessels to tightly regulate the movement of molecules, ions, and cells between the blood and the CNS [13] and ensure that the composition of the brain ISF is relatively independent of the composition of blood plasma. Like other tissues, the CNS's capillary bed is made up of ECs, which have TJs that significantly restrict the passage of chemicals and ions across cells (paracellular). Besides, these ECs limit the transcellular flow of molecules in addition to lacking fenestra and having extremely low rates of pinocytosis and transcytosis [14]. To maintain ionic equilibrium and homeostasis, ECs that make up the BBB are controlled by neighboring cells such as pericytes, astrocytes, neurons, and microglia [15]. They directly affect ECs by secreting soluble substances or interacting with proteins that form cell-to-cell contacts [16]. Every type of cell is essential to the growth and maintenance of the BBB. Pericytes, which are involved in the control of angiogenesis, vascular remodeling, vascular tone, and the formation and function of the BBB, cover the abluminal surface of the endothelium in an incomplete layer [17, 18]. Mice born with pericyte deficiencies exhibited increased BBB permeability through higher rates of endothelial transcytosis [19]. Moreover, endfeet of astrocytes fully cover the vascular surface and are linked to each other *via* gap junctions, as well as ensheathing synapses, nodes of Ranvier, and blood vessels. This enables them to sense and control blood vessel function and neuronal activity to control arterial contraction and dilation to regulate region-specific blood flow in response to neuronal activity [20]. In addition, ECs co-cultured with astrocytes had a BBB phenotype and better barrier function, including the production of junctional proteins like zonula occludens (ZO)-1 [21-23]. Additionally, macrophages that are located on the abluminal surface of the vessels, in the space between the vascular cells and the glial endfeet, provide immunological surveillance [24]. Generally, the neurovascular unit is made up of these neural cells, vascular cells, and extracellular matrix components; any compromise of the BBB may increase the risk of CE.

Three distinct barriers—the glycocalyx, the endothelium, and the extravascular compartment—were found to be responsible for the passive transport of both small and large hydrophilic molecules over the blood-brain barrier, according to research by Kutuzov *et al.* [25]. A glycocalyx, a layer rich in carbohydrates, covers the luminal surface of the endothelium. Its soluble components are in a dynamic equilibrium with the bloodstream and are crucial for preserving the integrity of the endothelial layer [26]. The initial BBB barrier, the negatively charged glycocalyx, serves as a charge/size-selective sieve to restrict the interaction of chemicals and cells with the endothelium [25]. In addition, the perivascular Virchow Robin space is a CSF-filled extension of the subarachnoid space in arteries bigger than capillaries, delimited by the endothelial basement membrane on the inside and a second glial basement membrane on the outside. It has been discovered that the Virchow Robin space follows penetrating arterioles into the brain parenchyma, becomes fenestrated towards the capillary bed, and finally vanishes at the level of brain capillaries, where the endfeet of astrocytes directly contact the vessel wall [10]. Damage to any part of these three distinct barriers of the brain can lead to impairment of the blood-brain barrier.

### 2.2 Mechanisms of Cerebral Edema

In this review, TBI is utilized as an example to explain how CE occurs. TBI frequently leads to neuronal apoptosis, either as a direct result of the trauma itself or as a consequence of hypoxia alterations, causing increased intracranial pressure or edema [27]. Along with the progression, as mentioned previously, CE could be identified as three distinct types, namely cytotoxic, interstitial, and vasogenic.

First and foremost, the hallmark of CytE, which is primarily brought on by cellular metabolic failure, is the buildup of extra fluid in the intercellular space while the BBB is still intact. In human subjects, it has been recorded as early as one hour after TBI [28]. Oxidative stress is one of the primary contributors to CytE. This stress causes brain cells to become injured, which in turn causes an imbalance of ions, which is the circumstance that leads to swelling. Recently, studies based on oxidative stress during TBI merit neuroscientists’ attention and provide novel light on the clinical treatment for TBI. For instance, a study first to experimentally examine the presence of Forkhead Box class O (FOXO) 3a expression in human brain tissues after TBI using immunohistochemistry analysis revealed that the expression of FOXO3a was upregulated following TBI. This study indicated that the expression of FOXO3a is increased in neurons due to oxidative stress generated by Reactive Oxygen Species (ROS) through a dual-signaling route [29]. It is believed that FOXO3a enhances cellular apoptosis, neuroinflammation, and the reaction to oxidative stress in tissue after TBI [29], therefore, the inhibition of FOXO3a might be a potential treatment for TBI. In addition, 8OHdG, a prominent type of DNA damage caused by ROS, which is commonly utilized as a highly dependable biomarker for the presence of oxidative stress, has been observed in the group of participants who died as a result of TBI, compared to subjects in the control group [27]. Furthermore, 3,4-Methylenedioxy-methamphetamine (MDMA)-induced neurotoxicity results in the creation of quinone metabolites and hydroxyl radicals, which subsequently generates ROS [30]. The investigation carried out by Riezzo *et al.* has shown that oxidative stress is the cause of MDMA-induced neurotoxicity, namely in the early phases [30]. As a result, we expected that reducing MDMA might further be a potential method for protecting CytE.

Moreover, CytE can manifest in various cell types within the CNS, with a particular predilection for astrocytes [31] and ECs. The outermost layer of the BBB is composed of a tightly interwoven mesh of astrocyte processes. Astrocytes, characterized by an endfeet membrane domain enriched in channels facilitating water and ion movements, are organized in a three-dimensional matrix of non-overlapping spatial domains, effectively occupying the entire brain parenchyma [32]. It takes the aquaporin-4 (AQP4) protein, which is exclusively expressed by astrocytes, to equilibrate osmotic gradients brought on by ionic transport [33]. The stimulation phase of potassium spatial buffering is when astrocyte swelling occurs due to AQP4, according to the findings of certain investigations in knockout mice [34, 35]. It is reported that AQP4 dysregulation becomes more uniformly distributed on the astrocyte plasmalemma and appears after injury compared to the perivascular localization of AQP4 in the healthy cortex [32, 36, 37]. Following injury, proteinase released by astrocytes and microglia probably leads to the loss of laminin, agrin, and β-dystroglycan from the basal lamina [36]. The loss of these perivascular anchors causes AQP4 to diffuse throughout the astrocyte membrane during a similar period as CytE, which implies that these phenomena may be related.

The Sulfonylurea Receptor 1 (SUR1) is an ion channel that is essential in ion homeostasis in the brain. It has been found that SUR1 is upregulated in many kinds of cells, such as oligodendrocytes, microglia, astrocytes, neurons and microvascular endothelial cells under ischemic conditions in brain tissue [38, 39]. Transient Receptor Potential Melatonin 4 (TRPM4) is upregulated concomitantly with SUR1 after cerebral ischemia, during which SUR1 binds to it to form SUR1-TRPM4, an octameric monovalent cation channel. This kind of channel was first identified in adult rat astrocytes [10, 40]. In addition, SUR1-TRPM4 can bind to AQP4 to increase cation and water influx into a variety of cells, including astrocytes. However, increased expression of SUR1 contributes to vascular injury and plays a role in angioedema, as evidenced by SUR1-TRPM4-mediated death of tumor cells involved in maintaining the structures of the BBB (*e.g*., astrocytes, endothelial cells), leading to capillary dissection, which exacerbates angioedema and can eventually lead to secondary hemorrhagic progression [40-43]. TRPM4 constitutes the pore-forming subunit of the channel and is a constitutively expressed monovalent calcium-sensitive cation channel [38, 44]. When co-assembled with SUR1, TRPM4 functions as a negative regulator of Ca2+ and is amplified [45]. If the channel is damaged, this will result in abnormal opening of the channel, allowing depolarization of cells due to Na^+^ influx, tissue swelling, hemorrhage, and cell necrosis [10, 31, 40, 45-48]. The SUR1 pathway has also been associated with other factors that affect blood-brain barrier integrity and promote brain edema, including tissue plasminogen activator, Nitric Oxide Synthase (NOS) 2, and Matrix Metalloproteinase (MMP)-9 [49-51].

Na^+^–K^+^–2Cl^-^ Cotransporter (NKCC) 1 is another important target to regulate the osmotic pressure of cells. It can transport a sodium ion, a potassium ion, and two chloride ions into cells in the same direction and enhance the osmotic gradient. Studies have shown that the up-regulation of the with-no-lysine kinase (WNK) protein family can be observed in brain injuries such as cerebral hemorrhage and ischemic stroke. Notably, WNK3 activates NKCC1 by phosphorylating SPAK, an important upstream regulator of NKCC1 [52]. The sustained opening of NKCC1 allows ions to enter cells in large quantities, inducing cellular swelling and CytE [52, 53]. In addition, NKCC1 is also involved in the joint action of AQP4. NKCC1/AQP4-mediated astrocyte swelling results in the infiltration of glutamate and other excitatory amino acids, the production of NO, and the release of cytokines such as Interleukin (IL)-1β, IL-6 and Tumor Necrosis Factor-alpha (TNF-α), damage to the BBB in turn exacerbates brain edema [54].

In Vasogenic Edema (VasE), macromolecules and water flow through dysfunctional BBB and accumulate in extracellular space, which leads to Cerebral Blood Flow (CBF) changes and ICP increase. The proteinaceous fluid influx may further raise colloid osmotic pressure in the extracellular space and cause local hypoperfusion, exacerbating CytE. We have previously mentioned that AQP4 is one of the portals to mediate CytE. In contrast, AQP4 has been reported to have apparadictory roles in VasE. As AQP4 knockout impairs mice’s ability to efficiently clear the edema, AQP4-dependent water efflux at the astrocyte endfoot appears to be responsible for the resolution of VasE [55], and thus AQP4 is now regarded as a rate-limiting factor in vasogenic edema formation.

Brain ECs maintain the low permeability of BBB through the basement layer rich in extracellular matrix proteins and TJs between cells. MMPs (which could be activated by oxidative stress) [56] are a group of proteases containing Zn^2+^ that can degrade extracellular matrix and are key molecules in vasogenic brain edema caused by BBB disruption [57]. MMP-9 can damage the BBB basement membrane by hydrolyzing collagen IV and laminin, resulting in leakage and promoting VasE [58]. In addition, a transmembrane glycoprotein called dystroglycan (DG), which is strongly expressed at the end of astrocytes, also connects the cell membrane to the basement membrane. According to Yan W’s study, MMP-2/MMP-9 mediated the cleavage of DG β subunit, resulting in BBB damage. Since DG disruption is associated with the abnormal polarization of AQP4, MMP-2/MMP-9 may further lead to CytE [59]. The TJ of BBB is composed of transmembrane proteins and cytoplasmic proteins on ECs. Occludin and Claudins are transmembrane components of TJ, while ZO-1 is the main cytoplasmic protein connecting transmembrane components and actin cytoskeleton [60]. The findings demonstrated that hypoxia enhanced the activity of MMP-9, and MMP-9 degraded Occludin and ZO-1 simultaneously, resulting in the interruption of TJ continuity and BBB leakage in mice [61].

Myosin light chain (MLC) is a calcium-binding protein involved in muscle movement. Phosphorylation of MLC by myosin light chain kinase (MLCK) causes contraction of actin in endothelial cells and increases the intercellular space between ECs, which leads to BBB leakage. In addition, MLCK can disrupt tight junctions by phosphorylating Occludin. Previous studies have shown that LPS stimulates the expression of MLCK through NF-κB pathway. It also induces the production of inflammatory mediators such as TNF-α and IL-1β, which disrupt BBB through direct oxidative stress injury and indirect MMP adjustment. What’s more, cyclooxygenase (COX) derivatives stimulate the expression of MMP-9/-3 to participate in BBB destruction and vasogenic edema. The mechanism is probably led by COX-2’s chemotaxis to neutrophils which contain large amounts of mature MMP-9, and COX-1’s activation to MMP-3 [62-65]. So, blocking the anti-inflammatory pathway is another important way to protect the integrity of BBB.

A polysaccharide structure called the glycocalyx, mostly made up of proteoglycans and glycoproteins, extends from a cell's body. In blood vessels, the vascular wall and the flowing blood are separated by the endothelial glycocalyx, which extends into the vascular lumen. In addition, the endothelium glycocalyx plays a role in modulating inflammatory responses, such as leukocyte rolling and extravasation, and controlling vascular permeability [66]. Nowadays, glycocalyx degradation can be observed in most brain injuries such as ischemic stroke, status epilepticus (SE) and cardiac arrest (CA). According to Pan's research, in CA rat models, the breakdown of the glycocalyx enhanced the permeability of the BBB, increasing the water content of both the hippocampus and cortex [67]. Furthermore, it is confirmed that glycocalyx degradation stimulates the upregulation of caveolae-mediated transcellular transport and induces BBB leakage [68]. With such ample experimental evidence, it can be said that glycocalyx plays an indispensable role in the formation of CE. Hydrocortisone (HC) and heparin showed magnificent effects in preventing glycocalyx loss and could be considered a promising strategy for edema protection after cerebral trauma [69]. Besides, as the main component of glycocalyx, hyaluronic acid has great hydration capacity. By enhancing the catabolism of hyaluronic acid, ischemic preconditioning may mitigate water entry into brain tissue [70].

### 2.3 The Update of the Glial Lymphatic System

CSF, as a protective fluid of CNS, can shield the brain and spinal cord from mechanical shock. Its secretion, flow, and reabsorption are highly related to the brain's nutrient supply and metabolic waste, as well as to the maintenance of intracranial pressure [71]. According to statistics, about 90% of cerebrospinal fluid is secreted by the choroid plexus. The cerebrospinal fluid secreted by the choroid flows from the lateral ventricle to the third ventricle through the interventricular foramen, then into the fourth ventricle through the aqueduct, and finally from the median foramen of the fourth ventricle to the subarachnoid space. Traditionally, CSF is considered to be absorbed from the arachnoid granules into the dural sinus and discharged directly into the venous blood. However, numerous investigations conducted recently have shown that some cerebrospinal fluid is indirectly discharged into venous blood through the lymphatic system, mainly through the cranial nerve sheath pathway and meningeal lymphatic vessels (MLV) [72]. An essential pathway along the cranial nerve sheath is the cribriform plate pathway, which extends from the olfactory nerve sheath to the lymphatic capillaries of the nasal mucosa [73]. In 2015, functional lymphatic MLVs in the dural sinus were confirmed by two experimental groups and were found to be the channels for transporting CSF and immune cells to the deep cervical lymph nodes (dCLN)s. Evans blue injected directly into the nasal mucosa did not reach the dCLN, according to Louveau's experiment, indicating that the predominant lymphatic drainage pathway in CSF should be MLVs rather than nasal mucosa lymphatics [74]. In contrast to the dorsal MLVs in the superior sagittal and transverse sinuses, which are tightly connected by a zipper pattern and lack lymphatic valves, basal MLVs are connected by the loose button-like lymphatic EC and are closer to the subarachnoid space in the anatomic location. Besides, the lack of smooth muscle around the lymphatic vessels and the presence of the lymphatic flap endows the basal MLVs with dual functions of macromolecule absorption and transportation [75]. MRI results imply that after injection of contrast medium into Cisterna Magna, the signal intensity peaks were observed in basal MLVs, dCLNs, and dorsal MLVs according to time sequence. It is suggested that basal MLV is the main pathway of CSF uptake and drainage in the meningeal lymphatic system [75].

Similar to peripheral lymphatic vessels, MLVs also express podophyllotoxin, Prospero homologous protein 1 transcription factor (Prox1), and VEGFR3. Members of the VEGF family and VEGFR tyrosine kinases control the growth and operation of the lymphatic and blood-vascular systems. Prox1 is the master regulator of lymphatic ECs that induce venous ECs into primordial lymphatic structures [77]. By using antibodies to a Green Fluorescent Protein (GFP) together with Prox1, Lymphatic Vessel Endothelial receptor-1 (LYVE-1) and Podoplanin as markers, Rebecca M.lzen found that MLVs develop during postnatal stages and extend alongside transverse sinuses and middle meningeal artery to superior sagittal sinus [77]. VEGFR3 interacts with only lymphangiogenic group VEGFs (VEGF-C and VEGF-D). VEGF-C, a ligand of VEGFR3, is essential for the generation and maintenance of meningeal lymphatics, while VEGF-D is dispensable. Meningeal lymphatics may regress as a result of the disruption of the VEGF-C/VEGFR3 signal transduction system caused by the use of VEGFR3 tyrosine-kinase inhibitors, VEGF-C D traps, or VEGF gene blockage [78]. In an experiment of inhibiting VEGFRs with sunitinib, the ability of meningeal lymphatics to regrow was observed after drug withdrawal, indicating that the growth of the meningeal lymphatics system has continuous plasticity [78]. Dorsal MLV ablation reduced the efficacy of anti-PD-1/ CTLA-4 combination therapy for GL261 tumors, while mice with tumors overexpressing VEGF-C consistently showed decreased tumor sizes and tumor weight, highlighting the critical role MLVs play in anti-tumor therapy [76].

ISF is the place where the brain parenchyma cells live directly and can dissolve the metabolic wastes in the brain parenchyma. CSF and ISF pass through the Perivascular Space (PVS), which is a space between the endothelium and the astrocytes around it. Although the brain lacks conventional lymphatic circulation, it does have an astrocyte system (the glial lymphatic system) that coordinates with the cerebral vascular system to ensure a mixed exchange of ISF and CSF. In this lymphatic system, fluid pressure from the CSF from the choroid and pulsation of the cerebral arteries push the CSF from the periarterial space deep into the brain. AQP4 aquaporins, which are polarized on the telopodia of astrocytes, enter the brain stroma, mix with ISF, and flow back into the PVS. Eventually, the CSF-ISF mixture travels to the brain *via* the meningeal lymphatics, cranial nerve sheaths, or nasal mucosa lymphatics [79]. In addition to the CSF-ISF exchange function, numerous studies have shown that the glymphatic system is at the core of the brain's clearance function, mainly mediated by astrocyte AQP4. The glymphatic solute clearance function was disrupted in AQP4-null mice, and pathological deposition of β-amyloid [80], Tau [81] and α-synuclein [82] were observed, which was clinically associated with the development of neurodegeneration. To conclude, the glial lymphatic system consists of three key compartments: a periarterial CSF influx route, a perivenous ISF efflux route, and a parenchymal exchange pathway dependent on astrocytic AQP4. The imbalance among them will trigger and exacerbate the formation of CE and hinder its resolution.

## 3 THE ROLE OF THE UPDATED GLYMPHATIC SYSTEM IN CEREBRAL EDEMA

### 3.1 Relationship between the Meningeal Lymphatic Vessels and Cerebral Edema

We have previously introduced a brief concept that a functional role played by the MLVs in the glymphatic system might be of great importance to drain CSF in CE.

Ischemic stroke, as well as TBI, the predominant causes of CytE, can severely disrupt the cerebral glymphatic drainage system [83, 84]. For instance, in a mouse model of acute ischemic stroke, CSF inflow was reduced in the ipsilateral cortex after 3 hours of middle cerebral artery occlusion, resulting in the impairment of the glymphatic system [85]. During this process, within minutes, the glymphatic system allows for the rapid flow of lymph into the brain parenchyma, which also functions as the source of edema fluid. Whereas, spontaneous arterial recanalization boosted glymphatic perfusion within 24 h [85]. In addition, multiple experiments have directly expounded the crucial part the meningeal lymphatic system plays in the activation of the processes of brain drainage and edema clearing after stroke. In the transient middle cerebral artery occlusion (tMCAO) model, Yanev *et al.* found that meningeal lymphatic vessels sprouted from an adjacent sinus into the anatomical area corresponding to stroke, revealing that meningeal lymphatic hypoplasia exacerbates stroke severity *via* increasing infarct size and causing sustained motor deficits [86]. In another study, meningeal lymphatic cells were reported to rapidly grow into the injured brain in a parenchyma zebrafish ischemic stroke model induced by photochemical thrombosis. Of note, these ingrown meningeal lymphatic vessels contributed to resolving CE and directing and supporting the growth of nascent blood vessels [87]. These lymphatic vessels entering the brain’s parenchyma undergo apoptosis and are cleared after cerebral vascular regeneration. The role of lymphangiogenesis in alleviating the progression of edema has been confirmed in myocardial infarction, as well [88]. Furthermore, TBI was discovered to trigger compromised meningeal lymphatic drainage that can last at least one-month post-injury, resulting from increased ICP. Simultaneously, pre-existing deficits in meningeal lymphatic function predispose the brain to cognitive dysfunctions, impaired neuroinflammation and subsequent brain trauma due to the accumulation of waste products in the parenchyma [84].

VasE occurs mainly in the case of subacute stroke, brain tumors and intracranial infections. Interestingly, CytE will develop into VasE in the later phases of diseases. For example, neuroinflammation triggered by ischemic stroke plays a key role in the breakdown of BBB, causing the dysfunction of the glymphatic system, which contributes to VasE formation, hemorrhagic transformation, and aggravated patient prognosis [89, 90]. Furthermore, the increase in the diameter of meningeal lymphatic vessels has been observed in various experimental models, including but not limited to the opening of the BBB [91, 92], which might reveal that the drainage of CSF *via* meningeal lymphatic vessels is connected with VasE. However, Semyachkina-Glushkovskaya *et al.* observed that the diameter of meningeal lymphatic vessels increased *via* optical coherence tomography only under the circumstance of BBB impairment [91], and the increased diameter of meningeal lymphatic vessels indicated the activation of meningeal lymphatic drainage function after stroke [92]. Therefore, boosting the growth of meningeal lymphatic vessels appears to be advantageous for both tissue healing and CE resolution.

### 3.2 Meningeal Lymphatic Vessels are Engaged in Meningeal Immunity

As is well recognized, one of the common causes of edema in peripheral conditions is decreased lymphatic system function, which generates a pathological buildup of protein-rich lymphatic fluid in the intercellular interstitium. Interestingly, it is observed that tracers injected into the brain parenchyma and ISF enter the CSF and then dCLNs [93]. Before the discovery of MLVs, cellular and soluble constituents of CSF were thought to elicit immune responses in CLNs by entering the lymphatic vessels in brain mucosa through the cribriform lamina [94]. However, nowadays, studies have confirmed that the functionality of meningeal lymphatic vessels not only lies in taking up and draining CSF but is also reflected in directly communicating with CLNs to regulate intracranial inflammatory processes [75, 95]. MLVs act as a bridge between the CNS and the peripheral immune system, but further research is required to understand how CLNs and MLVs contribute to the immunological response.

Immune response in the brain is a complicated process. Damage-associated molecular patterns (DAMPs), like as HMGB1, are released by necrotic cells after an ischemia insult and activated microglial cells [96, 97]. TNF-α, IL-1β, reactive oxygen species, and inducible NOS are among the significant proinflammatory mediators that the activated microglia emit later to enhance neuroinflammation [98]. Since the expression of inflammatory mediators and matrix metalloproteinases increased, reactive astrogliosis is induced, and the BBB is further disrupted [99], which further aggregates neural injury and enables neural antigens that were drained into CLNs to trigger the systemic immune responses and causes immune cells to migrate to the brain [100]. According to reports, when brain antigens are recognized by dendritic cells (DCs), they get activated and may then go to CLNs as antigen-presenting cells (APCs) to activate T cells that are specific to those antigens [101]. The findings of investigations conducted on animals demonstrated that DCs enter lymphatic channels *via* upregulating the CC-chemokine receptor 7 (CCR7) and interacting with the CC-chemokine ligand 21 (CCL21) produced by MLVs [76, 102]. However, in CCR7-deficient mice, T cells and DCs fail to drain into CLNs, but it is meningeal lymphatics that enable T cells to enter CLNs by the CCR7-CCL21 pathway [103]. Moreover, since B cells are activated by soluble antigens, which are coupled to B-cell receptors, brain antibodies are released [104]. Subsequently, the infiltrating immune cells in the brain then exert their pro- or anti-inflammatory effects after being delivered into the blood vessels *via* lymphatic channels along with activated effector T cells and brain antibodies [101]. In models of experimental autoimmune encephalomyelitis (EAE), CLN ligation and ablation of meningeal lymphatics are certified to block lymphatic draining function that prevents the interaction of T cells and APCs and the activation of T cells in deep CLNs, resulting in the reduced invasion of activated T cells into the brain and the amelioration of the EAE [103], which also supports the pivotal role of meningeal lymphatics play in the brain’s immune response and immune cells transportation.

By activating the lymphatic endothelium of CLNs, elevated VEGF-C levels in CSF have been shown to increase the number of pro-inflammatory macrophages in focal cerebral ischemia-prone rat models [105]. *In vitro*, by blocking VEGF-C/VEGFR3 signaling in lymphatic ECs, it is discovered that cytokine/chemokine expressions in superficial CLNs and pro-inflammatory macrophages in the ischemic area are significantly decreased. Finally, an apparent reduction of cerebral infarction volume was also recorded [105]. The meningeal lymphatics may experience structural and functional changes under ischemic stroke, and their integrity is crucial for recovery from stroke. The VEGFR3 signaling pathway is crucial for maintaining the integrity of the lymphatic vessels because of a larger cerebral ischemia volume and worse prognosis, which were observed in the VEGFR3^wt/mut^ mice that showed fewer meningeal lymphatic vessels compared to the wild-type mice [86]. Of note, activated lymphatic endothelium could stimulate macrophages to secrete a multitude of pro-inflammatory factors to improve immune injury and breakdown of the BBB. In addition, T and B cells, as well as a variety of factors such as neuropilin-2 [106], angiopoietins [107], BMP9-ALK1 [108], and DAMPs [109] in the CLNs, are also involved in the immune damage and the activation of lymphatic endothelium after stroke. In a mouse model of Glioblastoma Multiforme (GBM), ectopic VEGF-C expression enhances CD8 T cell priming in the draining deep CLNs and migration into the tumor as well as rapid GBM clearance, resulting in a long-lasting antitumor memory response. As is demonstrated by their data, by promoting lymphangiogenesis in the meninges, prophylactic VEGF-C therapy can elicit a potent and durable T cell-reliant immune response against brain neoplasms [110]. Shockingly, VEGF-C also stimulates the drainage of MLVs in aged mice whose lymphatic drainage system has certain obstacles, resulting in improved cognitive function [111]. Thus, the VEGF-C appears to be a potential immunotherapy target for CE. Furthermore, a clinical trial revealed that the TBI patients with improved conditions had significantly lower levels of VEGF on day 7 compared with higher VEGF levels on day 21, which indicated that the rise of VEGF exhibits an adverse effect on recovery from TBI 4-14 days after the injury while an advantageous effect is displayed 14-21 days after TBI [112]. This also demonstrated the dual role of lymphangiogenesis and might provide novel ideas for the treatment of CE.

### 3.3 Do Immune Responses Protect or Aggregate Cerebral Edema?

**Fig. (1):**
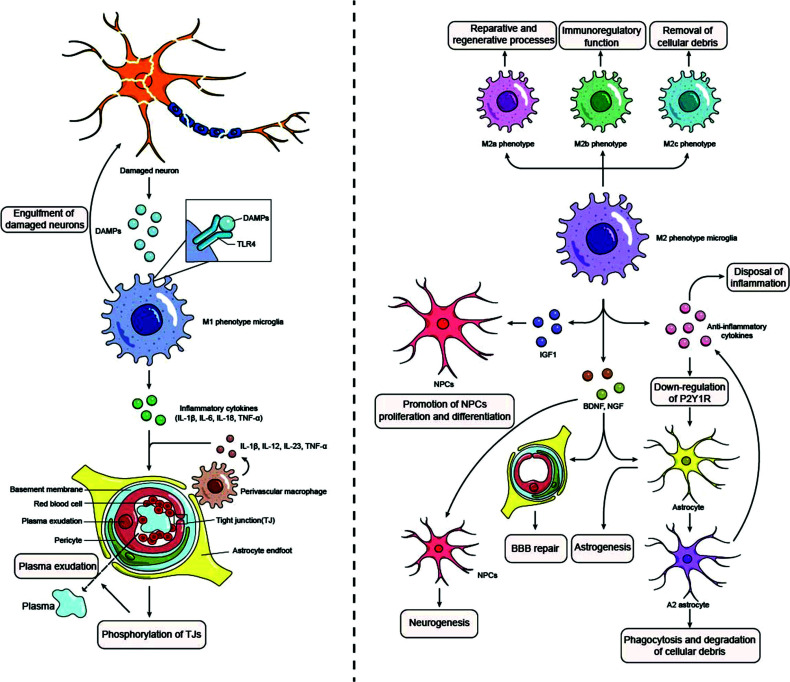
The paradoxical functions played by neuroinflammatory pathways. At different stages of cerebral ischemia, different phenotypes of microglia play different roles. Left: In the early phase of cerebral ischemia, M1 phenotype microglia produce proinflammatory cytokines that mediate the development of vasogenic edema. Right: In the subacute phase of cerebral ischemia, M2 phenotype microglia are activated to exert anti-inflammatory properties, which are manifested by the release of various cytokines that act on various cells in the brain tissue, such as neural precursor cells and astrocytes, to accelerate inflammation reduction and repair the blood-brain barrier.

The brain is an organ with “immune privilege” since immune cells from both the CNS and the periphery are enlisted to participate in inflammation responses following injury. Intravascular leucocytes are activated by stagnant blood flow and tissue trauma, and pro-inflammatory mediators secreted by the endothelium and parenchymal cells continue the inflammatory response, significantly amplifying tissue damage [113]. Nonetheless, the paradoxical functions played by neuroinflammatory pathways, which include mechanisms aiding reparative processes and improving healing in addition to being wholly destructive pathways, must nevertheless be understood [114, 115]. This would benefit us in our quest to comprehend the entire role of inflammatory responses in CE (Fig. **1**).

In the early stage of cerebral ischemia, damaged neurons emit DAMPs that induce Toll-like Receptor 4 (TLR4) on microglia in the early stages of cerebral ischemia, activating these cells to create inflammatory cytokines through the NF-κB pathway [57]. During this phase, the microglial of the M1 phenotype, believed to be pro-inflammatory, will produce pro-inflammatory cytokines such as IL-1β, IL-6, IL-18, and TNF-α [57]. Between the brain surface and the vascular endothelial membrane, perivascular macrophages secrete IL-1β, IL-12, IL-23, TNF-α, chemokines, and ROS [96], whose comprehensive function is to induce the phosphorylation of TJs [116], leading to BBB hyperpermeability and consequently initiates the occurrence of VasE. These biomarkers could reversely be detected to estimate the age of TBI, and further contribute to the diagnosis of CE. To show merely a few, the expression of TNF-α follows a distinct pattern in the ipsilateral choroid plexus. There are two peaks observed: an initial peak occurring 1 hour after trauma and a subsequent peak occurring 6 hours after trauma [117]. Additionally, in tMCAO mice, microglial and macrophage infiltration peaked at 48-72 h after the ischemic insult [118], then these cells migrated toward the ischemic lesion and remained close to the neurons in a mechanism known as “capping” to eliminate necrotic neurons rapidly [119, 120]. It was discovered that following TBI, there was a gradual rise in the quantity of CD68 cells (revealing a fundamental activation of macrophages and phagocytic activity) over a period of one to three days. Additionally, the morphology of these cells underwent a transformation, with an enlargement in size and a reduction in the length and thickness of their processes. CD68 exhibited the highest level of immunopositivity within or in close proximity to the necrotic regions at 15 and 30 days of survival [121]. Meanwhile, during the activation of M1 microglia, the clearance of glutamate by A1 astrocytes is interfered with the impaired expression of the excitatory amino acid transporter 2 (EAAT2) [122], while cytokines derived from neurons and glial cells lead to hyperplasia and activation of astrocytes [123]. These activated astrocytes release vimentin, IL-1β, monocyte chemotactic protein-1, and glial fibrillary acidic protein (GFAP), contributing to the formation of glial scars [124] as well as MMP-2, which leads to the impairment of BBB. What’s more, a systematic review illustrated an association between AQP4 and the accumulation of GFAP, a recognized indicator of TBI within the forensic domain [125]. Intriguingly, elevated levels of GFAP in several brain regions have been observed in response to large quantities of MDMA, which is mentioned as a biomarker of TBI before [30]. As a consequence, the neuroinflammatory response may be amplified by molecular interactions between reactive astrocytes and activated microglia [126]. Moreover, the cytokines and chemokines produced by microglia can also recruit leukocytes to the injured parenchyma to release proteases and oxygen radicals, thereby hastening tissue destruction [127]. The transition from CytE to VasE is aided by these intricate processes.

On the contrary, in the subacute phase, it is M2-polarized microglia that are activated and have anti-inflammatory properties [122]. Among them, M2a phenotypes are involved in reparative and regenerative processes, M2b phenotypes display immunoregulatory function characteristics, and M2c microglia remove cell debris [128]. Microglia release anti-inflammatory substances such as IL-4, IL-10, IL-13, or transforming growth factor-beta (TGF-β) once activated and polarized towards an M2 phenotype, contributing to the disposal of inflammation [129]. On the other hand, they produce insulin-like growth factor-1 (IGF1), which suppresses apoptosis and increases the proliferation and differentiation of Neural Precursor Cells (NPCs) [130], Brain-derived Neurotrophic Factor (BDNF), and Neuronal Growth Factor (NGF) to promote glial scar formation as well as BBB repairment, neurogenesis, astrogenesis, oligodendrogenesis, and angiogenesis [131, 132]. Meanwhile, by generating a range of neurotrophic factors throughout the chronic phase, M2-polarized microglia encourage neuroplasticity and neurogenesis [133]. Similar to microglia, the transition from A1 astrocytes into protective A2 phenotypes is stimulated by microglia-derived anti-inflammatory factors by downregulating the expression of P2Y1R in a brain trauma model [134]. However, more investigations are required in the future to shed light on the spatiotemporal dynamic and the mechanism underlying the activation and transformation of astrocytes. It is discovered that A2 astrocytes are qualified to secrete IL-2, IL-10, and TGF-β, leading to accelerated inflammation resolution. Pentraxin 3 is also released to attenuate IgG staining in ischemic cerebral tissue *via* the inhibition of VEGF [135]. Post-stroke BBB integrity and neurological function are protected by astrocytic IGF-1 by shifting immune cells to an anti-inflammatory profile in the ischemic environment [136]. Besides, under pathological situations, A2 astrocytes are capable of engulfing other cells: reactive astrocytes in the penumbra were found to swallow and digest cellular debris to do a favor for the resolution of inflammation *via* the ATP Binding Cassette A1 (ABCA1) after stroke [137]. Based on these complex mechanisms, astrocytes promote BBB repair by facilitating the resolution of inflammation, displaying their dual role in diverse phases. Intriguingly, the BBB is protected by Tregs acting on peripheral leucocytes in the early post-stroke phase by inhibiting neutrophil production of MMP-9 and preventing effector T cell activation by secreting anti-inflammatory IL-10 and TGF-β [138-140]. Five to seven days after the start of the stroke, Tregs infiltrate the brain parenchyma, drive microglial polarization toward the M2 phenotype, and inhibit astrocytic activation by blocking the Amphiregulin (AREG)/Epidermal Growth Factor Receptor (EGFR) pathway [141, 142]. Of note, Tregs encourage the proliferation of these cells in the subventricular zone as well as the migration of neural progenitor cells to the injured area and their differentiation into mature neurons by producing IL-10, which interacts with the IL-10 receptor expressed on neural stem cells [143]. Similarly, some cytokines also exhibit anti-inflammatory functions. For instance, IFN-β has been utilized to lower BBB disruption and MMP-9 levels [144, 145]. In a rat model of stroke, it was observed to decrease BBB breakdown, neutrophil infiltration and intercellular adhesion molecule (ICAM)-1 expression on cerebral endothelial cells, which reduced the size of the infarct [146, 147]. As is mentioned above, IL-4 aids in to transfer of microglia toward the M2 phenotype and indirectly supports cell survival and tissue repairment [129, 148].

Generally, in the case of CytE, inflammation responses play a role in destroying the integrity of BBB. However, the function of protection demonstrates that inflammatory reactions are not just the outcome of CNS cell injury but also defense mechanisms against worsening VasE.

### 3.4 Does Cerebral Edema Necessarily Mean Intracranial Hypertension?

CE contributes to an increase in intracranial volume, which is one of the causes of intracranial hypertension occurrence. The condition of CE has historically been associated with intracranial hypertension, and some pertinent criteria have been developed for determining CE by monitoring intracranial pressure. As we have already mentioned, the Monro-Kellie ideology links the advancement of CE to the operational standard of ICP. Whereas, in recent years, we have found that diffused CE primarily results in a global rise in ICP, but focal CE can develop into cerebral herniation syndromes with or without ICP elevation [5], manifesting that CE is not sufficient for intracranial hypertension. Therefore, ICP seems to be an inadequate representation of cerebral edema, which suggests that the necessary link between CE and ICP elevation does not exist, and thus, generalized treatment of ICP alone may not influence key pathophysiological mechanisms of cerebral edema.

A significant body of research also suggests that there is no required connection between the two. In a clinical trial embracing patients with hypertensive intracerebral hemorrhage (HICH), patients receiving the suction to remove the hematoma under keyhole endoscopy displayed a 10.9% rate of severe edema, compared to a rate of severe edema in large trauma craniotomy groups was 72.1%. However, both groups' ICP returned to normal when the hematoma was removed [149]. This outcome also shows that such an injury could not be fully resolved by a craniotomy and that CE may continue since the operation actually creates an outlet for brain tissue. Another study compared the results of cisternostomy with decompressive craniectomy and found that the former was more effective in lowering CE and ICP elevation [150]. Furthermore, the intracranial pressure in the study conducted by U. Ito *et al.* reflected the progression of brain swelling but was not particularly high during the presence of maximum edema [151]. A positive correlation between perihematomal edema reduction and the proportion of intracerebral hemorrhage (ICH) removed was represented, while this relation was unaltered by a dose of rt-PA, osmotherapy, or ICP therapy in multivariate analyses [152]. Additionally, the dynamics of the different substances, including an increase in ICP and glutamate concentration and lactate-pyruvate ratio was followed by massive edema and large infarcts, whereas in patients without progressive space-occupying infarcts, low and stable ICP and substrate concentrations were observed [153].

The requisition of converting CE into intracranial hypertension is hydrocephalus formation. It is acknowledged that due to the cells' hypoxia and nutrition starvation, mild cerebral ischemia primarily causes cytotoxic edema without intracranial pressure [154]. However, based on what we have mentioned above, we suppose that in addition to the location and severity of the ischemia, the brain's ability to protect itself also plays a role in why focal CE inexplicably results in intracranial hypertension. Consequently, from our point of view, ideal methods of addressing the issue of intracranial hypertension might not be considered as dream ways to resolve complicated CE.

## 4 POTENTIAL MANAGEMENT OF CEREBRAL EDEMA

### 4.1 Clinical Treatment for Cerebral Edema

As of now, mannitol and hypertonic saline—two typical pharmacologic agents—are employed most frequently in osmotherapy, the most popular approach to treating intracranial hypertension. It was not until that Wise and Chater found that mannitol had the benefits of longer ICP control, less “rebound overshoot” than urea, stability in solution, less risk of toxicity, and low cost in 1962 [155], mannitol became the recommended osmotic therapy agent of choice for decades. Hypertonic saline was gradually included for the treatment of increased ICP after rabbit research conducted in 1985 showed the cerebral effects of hypertonic saline on ICP and cerebral blood flow [156]. Mannitol, a freely filtered nonmetabolized sugar alcohol, is a potent osmotic diuretic that decreases the reabsorption of water and sodium across the renal tubules and has been widely used to treat elevated ICP. Additionally, for the past few decades, it has been regarded as a cornerstone in the medical care of CE. Nonetheless, mannitol only treats edema that develops in a non-ischemically injured area of the brain, and repeated infusions of mannitol have been suggested to exhibit CE aggravation if the osmotic substances migrate into the brain tissue through a disrupted BBB, reversing the osmotic gradient [157]. In a clinical trial, mannitol infusion improved brain relaxation in patients with midline shifts undergoing surgery for supratentorial tumors, although it was discovered that large doses of mannitol dramatically raised the incidence of moderate to severe CE [158]. This presumed adverse effect would be likely to occur with hypertonic saline as well. Notably, another downside of osmotic agents lies in the possibility of leading to dehydration and shrinkage of normal brain tissue and may facilitate displacement of brain tissue and even increase the risk of herniation [159]. There is an increasing number of studies certificating that it is not advisable to continuously provide osmotic medicines because of the risk of rebound cerebral edema, which poses extra risks to patients. Furthermore, frequent dosing could result in hyperchloremic metabolic acidosis, which has been associated with greater mortality in ICH patients, and faster administration of hypertonic saline has the potential to cause hypotension [160]. Nowadays, surgery is also regarded as an effective way to settle some severe cases of ICP elevation. To show only a few, for individuals whose ICP is elevated as a result of obstructive hydrocephalus, the implantation of an external ventricular drain (EVD) for CSF diversion is regarded as the first line of treatment. Decompressive surgery is preferred over global ICP-directed therapies for patients with increased ICP as a result of a localized compressive brain lesion. Decompressive craniectomy should be thought of as a third-line therapy for patients with intractable intracranial hypertension who have global or multifocal brain damage, such as severe TBI [161, 162]. However, as we have previously adverted, a craniectomy is likely to offer an outlet to brain tissue, which contributes to further CE. Therefore, it is imperative to present some promising molecular candidates that target the underlying pathophysiology of CE because there are considerable side effects associated with both osmotic medications and surgery. If these candidates are successful, doctors may be able to reduce the usage of nontargeted therapy.

### 4.2 Recent Researches Advance in the Glymphatic System

Since the discovery of MLVs in 2015, more researchers have been working in this field using a variety of models, such as cells, animals, and humans, contributing to the update of the glymphatic system. The findings of the MLVs to the glymphatic system have led to a more nuanced understanding of the exchange and circulation of CSF and ISF in the brain, doing us a favor to deeply understand the concept of metabolic waste drainage and brain immune privilege as well as the pathology of different neurological diseases, including neurodegenerative diseases, TBI, stroke and especially CE. In order to support the involvement of the glymphatic system in our study, we reviewed the literature for almost five years listed in Table **1**, looking forward to paving the way for further studies in this domain.

### 4.3 Potential Targets to Treat CE

**Fig. (2):**
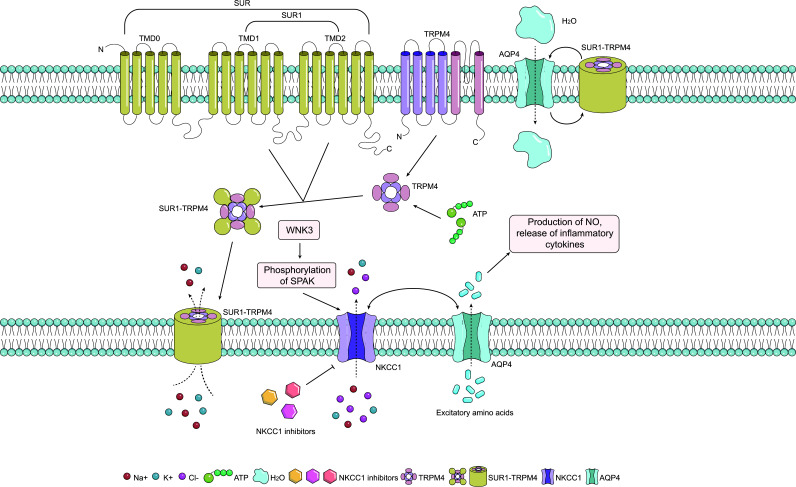
The position of different types of channels in water and ion transport and related potential targets for drug action. (**A**) SUR1 consists of two interacting transmembrane structural domains (TMD1 and TMD2). Four SUR1 subunits combine with four TRPM4 subunits to form a functional SUR1-TRPM4 octamer. SUR1-TRPM4 channels mediate the entry of Na^+^, K^+^, and Cl^-^ into the cell. The interaction of AQP4 and the SUR1-TRPM4 channel facilitates the penetration of water molecules into the cell. (**B**) WNK3 activates NKCC1 by phosphorylating the upstream regulator SPAK, promoting the transport of ions which are translocated into the cell. This function is inhibited by NKCC 1 inhibitors. The combination of NKCC1 with AQP4 accelerates the infiltration of excitatory amino acids, the production of NO, and the release of inflammatory cytokines. (**Abbreviations**: AQP4, aquaporin-4; NKCC 1, Na^+^–K^+^–2Cl^-^ cotransporter 1; NO, nitric oxide; SUR1, sulfonylurea receptor 1; TRPM4, transient receptor potential melatonin 4; TMD, transmembrane structural domains).

In this section, we will summarize emerging targets for CE treatment based on supporting molecular data discussed in Sections 2 and 3 (Fig. **2**), which are at various stages of development (Table **2**). However, whether these pharmacology agents could be used in future clinical practice still lacks adequate proof. We suppose that there are still concerted efforts to put in order to clarify the definite mechanisms as well as the feasibility of applying them to treat CE caused by diverse CNS diseases.

Glibenclamide is a second-generation sulfonylurea developed for type 2 diabetes targeting the SUR1-TRPM4 channel, and glibenclamide inhibits SUR1-TRPM4 channel upregulation after CNS damage. *In vivo* studies have noted that glibenclamide-mediated inhibition of the SUR1/TRPM4 reduces brain edema and brain death after ischemic brain injury [163]. Notably, glibenclamide may not be actively transported to brain tissue to produce effects, as glibenclamide, which is used clinically to lower blood glucose levels in diabetic patients, does not penetrate the intact BBB. Glibenclamide is leaked into brain tissue when caused by post-ischemia, leading to BBB rupture, and the drug gradually accumulates in the injured brain tissue. Meanwhile, to explore the therapeutic effect of high and low doses, it was found that it appears that low doses of glibenclamide can have a therapeutic effect on ischemia-related edema with minimal effect on blood glucose [41]. Previously, there were several teams developing potential therapeutic agents for CE based on glibenclamide that showed good therapeutic efficacy. One of them is that Deng and his team developed nanoparticles of betulinic acid from the Chinese herb Eucommia ulmoides, which can carry glibenclamide to effectively penetrate the BBB and also reduce brain infarct size and brain edema in mice. The nanosystem can target SUR1-TRPM4 and oxidation to reduce the effective dose of glibenclamide and improve the anti-brain edema efficacy of glibenclamide [164].

As we have previously mentioned, AQP4^–/–^ mice have reduced edema in models of relatively pure CytE while worse edema in models with predominantly VasE [33, 55]. A monoclonal AQP4-specific antibody called aquaporumab, has been utilized to lessen neuromyelitis optica pathology *in vivo*; however has not been evaluated in TBI [165]. On the other hand, no human research has been conducted. By blocking AQP4, thyroid hormone therapy also lowered CE and may be neuroprotective for stroke patients [166, 167]. Inhaling hydrogen sulfide (H_2_S) was found to preserve the BBB and minimize CE in rats [168]. The combination use of cerebrolysin and aquaporin inhibitors in a permanent-MCAO model also decreased the development of cerebral edema [169]. Importantly, although aquaporins have a role in CytE, inhibiting AQPs only works when the illness is still in its early stages [170]. The specific AQP4 inhibitors, AER-270 and AER-271 (a phosphorylated pro-drug of AER-270), are now being studied as potential agents in the control of CE in various models [171]. The intrinsically poor 'druggability' of AQPs as targets for small compounds and difficulties in creating and conducting reliable high-throughput screens may be contributing factors to the delayed progress in AQP inhibitor development.

Inhibited by the potent loop diuretic bumetanide [172], NKCC1 has been associated with CE development, and transporter activity is associated with neurotoxicity. NKCC1 is constitutively expressed on plasma membranes of multiple CNS cell types and is further upregulated within an hour of injury in preclinical TBI models [173]. Meanwhile, enhanced NKCC1 activity following brain injury may also contribute to upregulating AQPs, further compounding CE generation [174]. In TBI and spinal cord injury models, bumetanide boosts BBB integrity while lowering AQP4 expression, CytE, and neuronal death *in vivo* [175, 176]. Furthermore, in an *in vitro* experiment, bumetanide was observed to reduce astrocyte swelling in the TBI model [177]. However, bumetanide has not been tested on humans for TBI. Of note, the low permeability and existence of active efflux transport in the BBB will result in lower levels of bumetanide in the brain parenchyma (and CSF) than in other tissues [172].

VEGF is a significant proangiogenic peptide that is partly to blame for the loss of integrity of the BBB. The brain's endothelium is stimulated by VEGF to form interendothelial gaps, fragmentations, and fenestrations, which are linked to basement membrane degradation [178]. Due to fluid leakage from the intravascular compartment into the brain parenchyma brought on by these structural alterations, VasE and elevated interstitial fluid pressure are produced. It is believed that corticosteroids can reduce vascular permeability and so have an antiedema effect. VEGF is downregulated in part to produce this effect [179]. Corticosteroids help to lessen the signs and symptoms of VasE, but they also display several side effects, including hyperglycemia, gastrointestinal discomfort, sleeplessness, altered mental status, myopathy, and osteopenia. Consequently, alternative anti-VEGF treatments for CE are needed. In one trial, bevacizumab, a type of VEGF monoclonal antibody was given to patients with cerebral radiation necrosis, and later, the patients' contrast leaking and edema were significantly decreased [180]. Importantly, some patients were able to cut back on their corticosteroid dosage.

Results from hepatic encephalopathy (HE) rats provided direct evidence that lymphangiogenic factors generated in the brain, such as VEGF-D and IGF-1, may be transferred to the meninges and promote lymphangiogenesis. Interestingly, intra-cisternal injection of AAV-VEGF-C and control AAV-GFP to these rats resulted in a large increment in the expression of these genes in the meninges [181], which suggested that AAV-VEGF-C and AAV-GFP might have the power to treat CE targeting MLVs. In another study, increased lymphatic vessel diameter was detected in elderly mice aged 20-24 months administrated with AAV1-CMV-mVEGF-C without having any discernible off-target effects on the meningeal blood vasculature coverage and on meningeal and/or brain vascular hemodynamics [111]. Recombinant human VEGF-C increases dural lymphangiogenesis and eliminates both soluble and insoluble Aβ in Alzheimer’s disease (AD) models, which also provides fresh suggestions for AD treatments [182]. Conversely, these medications have not yet been used in a clinic and are still in the experimental stage. It is appealed that more clinical trials should be implemented and more MLV-targeting agents emerge in order to resolve the issues of CE arising after multiple neurological diseases in the future.

## CONCLUSION

CE, a complicated pathobiological phenomenon as well as a key determinant of secondary injury, occurs in various types of CNS insults. It is believed that if untreated, CE can be fatal, for that it is likely to induce intracranial hypertension, which will pose a risk to patients’ lives. However, in this review, we shed novel light on the relationship between CE and elevated ICP with the backing of clinical evidence: given the possible self-protection mechanisms in the brain, intracranial hypertension may not necessarily be the ultimate outcome of CE. It was not until the MLVs were detected in rodents that our understanding of CE began to shift from the BBB to the updated glial lymphatic system embracing meningeal lymphatic vessels. This discovery aids in interpreting molecular and cellular mechanisms of preventing CE from aggregated progression based on a potential dual role. In the initial stages of edema production, the glymphatic system is detrimental, but after edema subsides, it serves a helpful purpose.In addition, the function of astrocytes in edema should not be underestimated because they not only support the glymphatic system *via* AQP4 but also get in the act of immunological responses. Up-regulation of AQP4 can worsen the development of brain edema under cytotoxic edema conditions, while during vasogenic edema, it is crucial for fluid expulsion. Last but not least, as CE progresses, different phenotypes of microglia and astrocytes also exhibit dual inflammatory and anti-inflammatory effects. To ascertain whether the universality of this finding is contingent on the types of diseases that induce edema, a great deal of more research will be required.

The landscape of CE diagnosis and management is currently evolving in light of the new notion of the glymphatic system. In addition to current reactive practices, we discuss some molecularly derived approaches by targeting important pathways/contributors that have been studied in recent years, such as drugs targeting NKCC1, AQP4, MLCK, SUR1-TRPM4, VEGF, and so on, whereas there are probably still a great number of them to be discovered. The present study has also reported that routine surgical procedures, including implantation of EEG electrodes and chronic cranial window implantation, can induce meningeal lymphangiogenesis and increase glymphatic CSF circulation [183], which provided novel ideas for surgical treatment of CE as well as other neuroinflammatory diseases like AD. In addition, a new niche of immunotherapy for peritumoral edema is opened by the combination of photodynamic therapy with BBB opening, which is related to the stimulation of lymphatic drainage and clearing of the brain tissues [184]. Moreover, other alternative modalities involving recombinant proteins and various gene-based therapies are being developed, but they are currently in their infancy with regard to clinical translation [185]. In the future, it is anticipated that these techniques will become sufficiently mature to be gradually implemented in clinical practice alongside other physical treatments containing electronic pulse and rehabilitation therapy, which could be seen as a promising development for improving the prognosis of patients with CE.

## Figures and Tables

**Table 1:** Recent findings of meningeal lymphatic vessels.

**Models**	**Findings**	**References**	**Author (Year)**
EAE mice	(1) CCR7 controls immune cell entry into draining lymph nodes *via* meningeal lymphatics. (2) T cells interacting with antigen-presenting cells are reduced when meningeal drainage is reduced. (3) Inflammation does not cause meningeal lymphatic ECs to expand, and their transcriptional signature is unique.	[103]	Antoine Louveau *et al.* 2018
EAE mice	Lymphangiogenesis occurs in meningeal lymphatics next to the cribriform plate rather than in MLVs during EAE, suggesting the CNS lymphatics are heterogeneous.	[186]	Martin Hsu *et al.* 2019
Mice	(1) An electroencephalogram recording shows a positive correlation between lymphatic influx and delta power, while a negative correlation exists between delta power and heart rate. (2) The glymphatic function is inhibited by isoflurane alone, while the glymphatic function is promoted by 2-agonist dexmedetomidine.	[187]	Lauren M. Hablitz *et al.* 2019
Brain vascular injury in zebrafish	BLECs quickly invade brain parenchyma in response to brain vascular injury, forming lymphatic vessels that resolve edema and promote cerebrovascular regeneration.	[87]	Jingying Chen *et al.* 2019
Mice	(1) Compared with dorsal MLVs, basal MLVs are the major routes for CSF macromolecular uptake and drainage. (2) It may be associated with impaired CSF clearance in elderly individuals that basal MLVs undergo lymphedematous changes with age.	[75]	Ji Hoon Ahn *et al.* 2019
Glioblastoma mice	(1) In the draining deep cervical lymph nodes, VEGF-C enhances the priming of CD8 T cells, the migration of CD8 T cells into the tumor, the rapid clearing of the glioblastoma, and the formation of long-lasting antitumor memory. (2) In addition to checkpoint blockade therapy, transfection of an mRNA construct expressing VEGF-C eradicates existing glioblastomas.	[188]	Eric Song *et al.* 2020
Mice and Zebrafish	An outstanding ability to internalize macromolecules in the CNS was found in LLECs, a novel mammalian cell type and can be mediated by VEGFR signaling.	[189]	Shannon Shibata-Germanos *et al.* 2020
Intracranial tumors mice	(1) Combined anti-PD-1/CTLA-4 checkpoint therapy in striatal tumor models was significantly reduced when dorsal MLVs alone were disrupted without affecting either basal MLVs or nasal LVs. (2) VEGF-C potentiates checkpoint therapy via the CCL21/CCR7 pathway, resulting from that up-regulating VEGF-C displayed a better response to anti-PD-1/CTLA-4 combination therapy, which was abolished by CCL21/CCR7 blockade, suggesting that.	[76]	Xueting Hu *et al.* 2020
TBI mice	(1) Increasing ICP can negatively affect meningeal lymphatic function. (2) With viral delivery of VEGF-C, elderly mice are prevented from developing gliosis following TBI by regaining meningeal lymphatic drainage.	[84]	Bolte *et al.* 2020
Postmortem human brain samples	In perivascular/perineural spaces throughout the brain, CD3+ T cells appear to be close to lymphatic marker-positive cells.	[190]	Éva Mezey *et al.* 2021
5xFAD mice	(1) Microglial inflammation is exacerbated, and AD emerges when meningeal lymphatic drainage is impaired. (2) The administration of VEGF-C to patients improved the clearance of Aβ by monoclonal antibodies.	[191]	Sandro Da Mesquita *et al.* 2021
EAE mice	(1) As a result of neuroinflammation, meningeal lymphatics along the cribriform plate undergo lymphangiogenesis in order to drain excess fluid. (2) An immune-regulatory niche exists within inflamed lymphatics, where CD11c+ and CD4 T cells capture and present antigen, thereby regulating neuroinflammation through an underappreciated interface.	[192]	Martin Hsu *et al.* 2022
Cephalitis mice	(1) MLV expansion is promoted by neurotropic viruses, but macromolecule drainage is impaired as well. (2) The presence of VEGF-C pretreatment promotes the expansion of MLVs and alleviates the effects of viral infections.	[85]	Xiaojing Li *et al.* 2022
SCVS mice	(1) Sub-chronic variable stress impairs the function of the meningeal lymphatics in female mice rather than in male mice as a result of alterations in their transcriptional profile. (2) Stress susceptibility is reduced in female mice by improving meningeal lymphatics.	[193]	Weiping Dai *et al.* 2022
Mice	(1) Flow velocity of periarterial CSF and arterial dilation/constriction are directly correlated. (2) Flow of lymphatic fluid was enhanced in the absence of neural activation by optogenetic manipulation of vascular smooth muscle cells. (3) Inflow into the lymphatic system is directly influenced by the dynamic changes in arterial diameter.	[194]	Stephanie Holstein-Rønsb *et al.* 2023
SAH mice	(1) SAH triggers MLVs impairment. (2) Inhibition of STAT3/Bcl-2 signaling by THBS1-CD47 interaction after SAH promoted MLEC apoptosis, thus affecting MLV function.	[195]	Xiaoyu Wang *et al.* 2023
Mice	In the cerebral vasculature, ultrasonication of circulating microbubbles causes volumetric expansion and contraction of microbubbles, which creates a microbubble pumping effect that can enhance encephalic lymphatic transport.	[196]	Dezhuang Ye *et al.* 2023

**Abbreviations**: AD, Alzheimer’s disease; BLECs, brain lymphatic endothelial cells; CNS, central nervous system; CSF, cerebrospinal fluid; EAE, experimental autoimmune encephalomyelitis; ICP, intracranial pressure; LLECs, leptomeningeal lymphatic endothelial cells; MLEC, meningeal lymphatic endothelial cell; MLV, meningeal lymphatic vessel; SAH, subarachnoid hemorrhage; SCVS, sub-chronic variable stress; TBI, traumatic brain injury; VEGF, vascular endothelial growth factor; 5xFAD mice, a mouse model of amyloid deposition that expresses five mutations found in familial AD).

**Table 2:** Emerging targets for CE treatment.

**Pharmacology** **Agents**	**Targets**	**Models**	**Relevant Mechanisms**	**References**
Bumetanide	NKCC1 inhibitor	(1) Astrocyte swelling *in vitro* model of FPI (2) TBI mice (3) Spinal cord injury mice	Bind inside the translocation cavity of NKCC1 to inhibit it from an extracellular location.	[172, 175-177]
Glibenclamide	SUR1-TRPM4 Inhibitor	(1) Freshly isolated type R1 astrocytes from adult rat brain (2) Humans	Affects SUR1 kinetics rather than blocking the channel pore directly.	[48, 197]
Aquaporumab	AQP4 monoclonal antibody	-	-	-
AER-270	AQP4 inhibitor	Water intoxication mice	Inhibits AQP4.	[171]
AER-271	AQP4 inhibitor	MCAO mice	Inhibits AQP4.	[171]
Amiloride	Mechanogated membrane ion channels inhibitor	TBI mice	Blocks the Na^+^ channel, the Na^+^/H^+^ antiport, and the Na^+^/Ca^2+^ exchanger.	[198-201]
Gentamicin			Inhibits Ca^2+^ channel TRPV5.	
Gadolinium			Alters the packing and lateral pressure of anionic lipids.	
Heparin	Glycocalyx	SE mice	Ameliorates glycocalyx degradation.	[202]
SR 49059	V1a receptor antagonist	TBI mice	Competitively inhibits V1a receptor.	[203, 204]
ML-7	MLCK inhibitor	TBI mice	Inhibits MLCK phosphorylation.	[205]
SB-3CT	MMP-2/9 inhibitor	MCAO mice	Alters their structures	[206, 207]
Fenofibrate	PPAR-α agonist	TBI mice	Reduces ICAM-1, iNOS (NOS2), MMP-9, and markers of oxidative stress.	[208, 209]
Pioglitazone/ Rosiglitazone	PPAR-γ agonist	TBI mice	Reduces IL-6, MCP-1 and ICAM-1.	[210]
Curcumin	Multiple targets	(1) SAH mice (2) HIBD mice (3) Humans	(1) Upregulates tight junction proteins (ZO-1, occludin, claudin-5), upregulates glutamate transporter-1 (2) Inhibits ICAM-1/VCAM-1, inflammatory cytokines (IL-1β, IL-6, TNF, NF-κB) and microglial activation (3) Downregulates VEGF, AQP4 and MMP-9	[211-215]
VEGI	VEGF inhibitor	TBI mice	(1) Reduces tissue loss and microgliosis. (2) Upregulates tight junction proteins (claudin-5, ZO-1, and occluding)	[216]
Bevacizumab	VEGF monoclonal antibody	Humans	Relieves glioblastoma peri-tumoral edema.	[217]
AAV8-VEGF-C	Meningeal lymphangiogenesis	HE mice	(1) Increases meningeal lymphangiogenesis (2) Reduces microglia activation	[181]
AAV1-CMV- mVEGF-C	Meningeal lymphangiogenesis	FAD mice	Increases meningeal lymphangiogenesis	[111]
Recombinant VEGF-C	Meningeal lymphangiogenesis	(1) APP/PS1 transgenic mice (2) AD mice	Increases meningeal lymphangiogenesis	[182]
MAZ51	CLNs	FCI mice	(1) VEGFR-3 tyrosine kinase inhibitor (2) Reduces inflammatory factors (CCL28, CCL2, TNF-α, IL-1β) and brain injury	[105]

**Abbreviations**: AD, Alzheimer’s disease; AQP4, aquaporin-4; CLN, cervical lymph nodes; FCI, focal cerebral ischemia; FPI, fluid percussion injury; HE, hepatic encephalopathy; HIBD, hypoxia-ischemic brain damage; MCAO, middle cerebral artery occlusion; NKCC1, Na^+^–K^+^–2Cl^-^ cotransporter1; NOS, nitric oxide synthase; SUR1, sulfonylurea receptor 1; SE, status epilepticus; SAH, subarachnoid hemorrhage; TBI, traumatic brain injury; TRPM4, transient receptor potential melatonin 4; VEGF, vascular endothelial growth factor.

## LIST OF ABBREVIATIONS

ABCA1: ATP Binding Cassette A1
AD: Alzheimer’s Disease
APC: Antigen Presenting Cell
AQP4: Aquaporin-4
AREG: Amphiregulin
BBB: Blood-brain Barrier
BDNF: Brain-derived Neurotrophic Factor
CA: Cardiac Arrest
CBF: Cerebral Blood Flow
CCL: CC-chemokine Ligand
CCR: CC-chemokine Receptor
CE: Cerebral Edema
CLN: Cervical Lymph Node
CNS: Central Nervous System
COX: Cyclooxygenase
CSF: Cerebrospinal Fluid
CytE: Cytotoxic Edema
DAMP: Damage-associated Molecular Pattern
DC: Dendritic Cell
dCLN: Deep Cervical Lymph Node
DG: Dystroglycan
EAAT2: Excitatory Amino Acid Transporter 2
EAE: Experimental Autoimmune Encephalomyelitis
EC: Endothelial Cell
EGFR: Epidermal Growth Factor Receptor
EVD: External Ventricular Drain
GBM: Glioblastoma Multiforme
GFAP: Glial Fibrillary Acidic Protein
GFP: Green Fluorescent Protein
HC: Hydrocortisone
HE: Hepatic Encephalopathy
HICH: Hypertensive Intracerebral Hemorrhage
ICAM: Intercellular Adhesion Molecule
ICH: Intracerebral Hemorrhage
ICP: Intracranial Pressure
IGF1: Insulin-like Growth Factor-1
IL: Interleukine
ISF: Interstitial Fluid
LYVE-1: Lymphatic Vessel Endothelial Receptor-1
Mfsd2a: Major Facilitator Superfamily Domain Containing 2a
MLC: Myosin Light Chain
MLCK: Myosin Light Chain Kinase
MLV: Meningeal Lymphatic Vessel
MMP: Matrix Metalloproteinase
NGF: Neuronal Growth Factor
NKCC: Na^+^–K^+^–2Cl^-^ Cotransporter
NOS: Nitric Oxide Synthase
NPC: Neural Precursor Cell
Prox 1: Protein 1 Transcription Factor
PVS: Perivascular Space
ROS: Reactive Oxygen Species
SE: Status Epilepticus
SUR1: Sulfonylurea Receptor 1
TBI: Traumatic Brain Injury
TGF-β: Transforming Growth Factor-beta
TJ: Tight Junction
TLR4: Toll-like Receptor 4
tMACO: Transient Middle Cerebral Artery Occlusion
TNF-α: Tumor Necrosis Factor-alpha
TRPM4: Transient Receptor Potential Melatonin 4
VasE: Vasogenic Edema
VEGF: Vascular Endothelial Growth Factor
WNK: With-no-lysine Kinase
ZO: Zonula Occluden
